# The Effects of Tail Biopsy for Genotyping on Behavioral Responses to Nociceptive Stimuli

**DOI:** 10.1371/journal.pone.0006457

**Published:** 2009-07-30

**Authors:** Maria Elena P. Morales, Robert W. Gereau

**Affiliations:** Washington University Pain Center and Department of Anesthesiology, Washington University School of Medicine, St. Louis, Missouri, United States of America; Sapienza University of Rome, Italy

## Abstract

Removal of a small segment of tail at weaning is a common method used to obtain tissue for the isolation of genomic DNA to identify genetically modified mice. When genetically manipulated mice are used for pain research, this practice could result in confounding changes to the animals' responses to noxious stimuli. In this study, we sought to systematically investigate whether tail biopsy representative of that used in standard genotyping methods affects behavioral responses to a battery of tests of nociception. Wild-type littermate C57BL/6J and 129S6 female and male mice received either tail biopsies or control procedural handling at Day 21 after birth and were then tested at 6–9 weeks for mechanical and thermal sensitivity. C57BL/6J mice were also tested in the formalin model of inflammatory pain. In all tests performed (von Frey, Hargreaves, modified Randall Selitto, and formalin), C57BL/6J tail-biopsied animals' behavioral responses were not significantly different from control animals. In 129S6 animals, tail biopsy did not have a significant effect on behavioral responses in either sex to the von Frey and the modified Randall-Selitto tests of mechanical sensitivity. Interestingly, however, both sexes exhibited small but significant differences between tail biopsied and control responses to a radiant heat stimulus. These results indicate that tail biopsy for genotyping purposes has no effect on nocifensive behavioral responses of C57BL/6J mice, and in 129S6 mice, causes only a minor alteration in response to a radiant heat stimulus while other nocifensive behavioral responses are unchanged. The small effect seen is modality- and strain-specific.

## Introduction

With the increasing use of genetically modified mice in the study of nociceptive processing, the practice of genotyping has become an integral component of pain research. In order to obtain DNA for genetic analysis, typically, a small piece of tissue is removed from the mouse, as with a tail biopsy, an ear punch, or a toe clip, by 3 weeks after birth. Although this is a common practice, the resulting impact on behavioral assays of nociception is unknown.

Mounting evidence indicates that noxious insult in early life can significantly alter nociceptive processing in adulthood [1]–[7]. Injury to the nervous system during the postnatal sensitive period of development can significantly alter the nociceptive circuitry, thereby inducing long-lasting changes in nociceptive thresholds. The polarity of this change is directly dependent on the type of injury and the sensory modality in question, with certain injuries leading to increased sensitivity [1]–[3], [7] and others resulting in decreased sensitivity in adulthood [4]–[7]. Removal of a small piece of the tail in young mice could present the type of insult that might alter behavioral thresholds in adulthood. Studies of early-life injury and nociception in adulthood have focused on injuries restricted to the hind paws or viscera, and have not yet examined the consequences of injury to the tail, likely the most common early-life injury experienced by laboratory mice.

One study has sought to identify the impact of tail amputation on behavioral responses to noxious stimuli. Zhuo [8] reported NMDA-receptor dependent changes in behavioral responses to noxious stimuli in tail-amputated mice as he observed significant differences in responses to the cold plate, hot plate and tail flick tests as well as decreased thresholds to mechanical stimulation of the tail using von Frey filaments. Although these data are striking, the methods used in this study are not representative of standard genotyping protocols and are more representative of significant tail amputation. In the Zhuo study, a 2.5 cm segment of the mouse tail was removed at 4–6 weeks of age; this injury is significantly different from that induced by tail biopsy for genotyping.

Standard genotyping protocols recommend biopsy of a minimal amount of tissue not to exceed 10 mm prior to 4 weeks of age, the point at which the tail has completely ossified (http://oacu.od.nih.gov/ARAC/Genotyping.pdf). In order to systematically examine the effects of tail biopsy for the purposes of genotyping on subsequent behavioral responses to noxious stimuli, we tested adult mice in which tail biopsies had been performed on P21 at weaning. We assessed hind paw responses to radiant heat and von Frey filament stimulation as well as tail withdrawal responses to mechanical pressure. In addition, behavioral responses to an inflammatory stimulus were recorded after formalin injection into the hind paw.

## Methods

### Animals

All experiments were performed in accordance with the guidelines of the National Institutes of Health and The International Association for the Study of Pain and were approved by the Animal Care and Use Committee of Washington University School of Medicine. C57BL/6J (original source: The Jackson Laboratory; Bar Harbor, ME) and 129S6 (129S6/SvEvTac, original source: Taconic; Hudson, NY) mice were bred in the Washington University animal housing facility. Animals were maintained on a 12 h light/dark cycle with *ad libitum* access to food and water. On P21, pups were weaned and tail biopsies performed. In the tail-biopsied group, a 5 mm segment from the tip of the tail was removed using a razor blade while the untreated group was handled similarly without tail biopsy. Beginning at 6 weeks after birth, animals were subjected to a battery of behavioral tests of nociception (for review see [9], [10]).

### Mechanical Sensitivity Test

Mechanical sensitivity was measured using calibrated von Frey filaments as previously described [9]–[13]. Briefly, animals were placed in Plexiglas cubicles on a wire grid on a raised platform in a room with constant white noise emitted from a noise generator. Animals were allowed to acclimate for at least 2 h before testing. Withdrawal thresholds were determined by stimulation of the plantar surface of the hind paw with von Frey filaments (North Coast Medical, Inc.; Morgan Hill, CA) of increasing bending force. The force at which the animal withdrew in response to 3 out of 5 stimulations was recorded as the withdrawal threshold. The test was repeated 3–5 times with 15 min between testing the same paw. The average of withdrawal thresholds from all repetitions was calculated for each animal.

### Thermal Sensitivity Test

Thermal sensitivity was measured as previously described [13], [14] using a modified version of the original Hargreaves test [15]. Animals were placed in Plexiglas cubicles on a heated glass plate maintained at 30°C and allowed to acclimate for at least 1 h. Using a 390G Plantar Test Apparatus (IITC Life Science Inc.; Woodland Hills, CA), radiant heat of a constant intensity (25% active intensity for 129S6 mice and 15% active intensity for C57BL/6J mice due to their differing sensitivity in this test [16]) was applied to the plantar surface of the hind paw. The withdrawal latency was determined as the time from stimulus initiation until paw withdrawal with a 20 s cutoff to avoid tissue damage. The test was repeated 5 times for each paw with 15 min between testing the same paw. The average of the withdrawal latencies for all repetitions was calculated for each animal.

### Tail Mechanical Sensitivity Test

Tail mechanical sensitivity was measured using a modification of the Randall-Selitto test [17] as previously described [11], [18]. At least 15 min after thermal sensitivity testing, the animals' tails were tested for mechanical sensitivity using an Analgesy Meter (Ugo Basile; Schwenksville, PA). Using a weighted lever, the apparatus applies increasing pressure to tissue positioned between the testing platform and a cone attached to the lever. The animal was gently wrapped in cloth and laid flat on a box adjacent to the testing platform. Efforts were made to minimize stress to the animals during handling, but the restraint itself is likely a source of stress that could modify behavioral output. The testing platform was lined with clay to form a wedge in which the tail was placed. Subsequently, the tail was positioned such that the cone was ∼1 cm from the base of the tail and the increase in pressure was initiated. The withdrawal threshold was determined by the force at which the animal vocalized, struggled, or moved the tail. The test was repeated 3 times with 15 min between tests.

### Formalin Test

In the formalin test [19], spontaneous responses to formalin injection were measured as previously described [13], [20]. C57BL/6J tail-biopsied and control mice were placed in testing chambers for at least 2 h of acclimation prior to injection. Formalin (2%, 10 µl; Sigma, St. Louis, MO) was injected into the right hind paw, and the time spent licking, lifting, and flinching was measured in 5 min blocks for 1 h after injection.

### Statistical Analysis

Data were compared using Student's t-test of group means for the mechanical, thermal, and tail mechanical sensitivity tests, and using a two-way repeated measures ANOVA for the formalin test. A p-value<0.05 was considered significant. All statistical analyses were conducted using Graphpad Prism 5 software (La Jolla, CA).

## Results

### Sex differences in behavioral sensitivity

As has been addressed previously in the literature, sex of the subjects can have a significant impact on behavioral measures of nociception [21]–[23]. In light of this effect, in the present study data have been separated by sex. Direct analysis of data by sex (two-way ANOVA) revealed a main effect of sex in thermal sensitivity of both C57BL/6J and 129S6 strains (p<0.01 for both strains) with females exhibiting shorter withdrawal latencies than males. In addition, in the 129S6 strain, a main effect of sex was observed in mechanical sensitivity of the paw (p<0.01) with females again exhibiting lower withdrawal thresholds than males. Interestingly, no such difference was observed in the C57BL/6J strain in the same test (p = 0.5845). Similarly, in the modified Randall Selitto test of tail mechanical sensitivity, a main effect of sex was not observed in either strain tested. Finally, in the formalin test, there was a main effect of sex in C57BL/6J mice (p<0.0001) with females spending more time exhibiting nocifensive behavior than males. In all tests, where a main effect of sex is observed, female mice exhibit more sensitivity than male mice.

### Mechanical sensitivity of the hind paws

**Figure 1:**
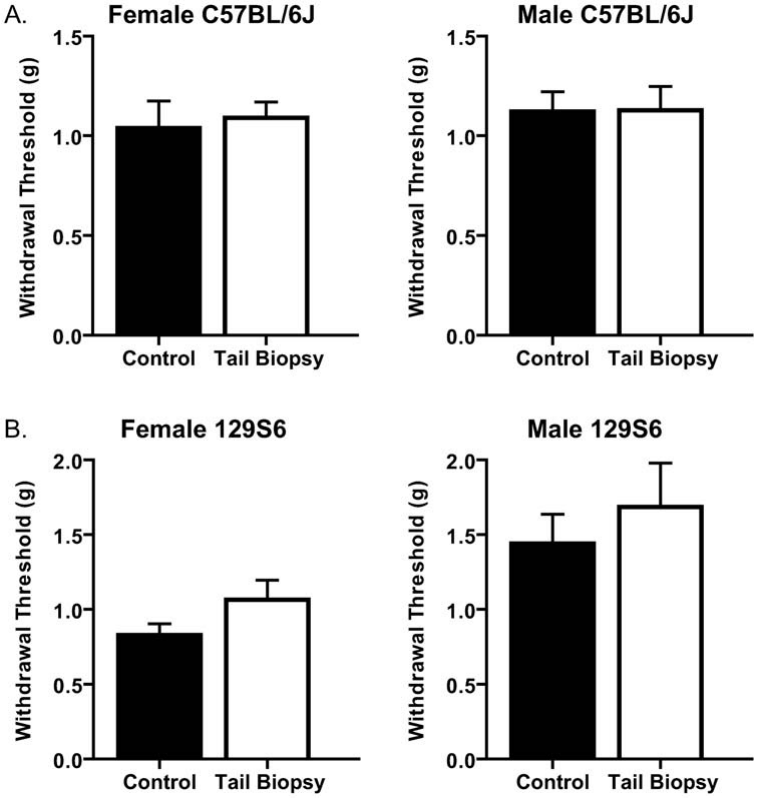
The effects of tail biopsy on mechanical sensitivity. There are no significant differences in mechanical sensitivity of tail-biopsied and control female and male mice of both C57BL/6J and 129S6 strains. A. Withdrawal thresholds (g) of tail-biopsied and control female (left panel, n = 12 in both groups; age range: 6–6.7 w) and male C57BL/6J mice (right panel, n = 14 tail biopsy and n = 13 control; age range: 6–6.7 w) do not differ (p = 0.7488 and 0.9677, respectively). B. Withdrawal thresholds (g) of tail-biopsied and control female mice (left panel, n = 8 tail biopsy and n = 11 control; age range: 5.71–7.14 w) and male 129S6 mice (right panel, n = 12 tail biopsy and n = 10 control; age range: 6–7.14 w) also do not differ (p = 0.1096 and 0.5148, respectively).

One of the most commonly used tests of sensitivity to mechanical stimuli examines withdrawal responses to von Frey filaments of increasing bending force. Although these responses were not directly examined by Zhuo (1998), the significant plastic changes he described could result in alterations in responses to these stimuli. In determining the effect of tail biopsy on these responses, we observed no significant differences in mechanical sensitivity of C57BL/6J female or male mice in response to hind paw stimulation using von Frey filaments (Fig. 1a). In addition, no significant differences were observed between tail-biopsied and control 129S6 mice of both sexes (Fig. 1b).

### Thermal sensitivity of the hind paws

**Figure 2:**
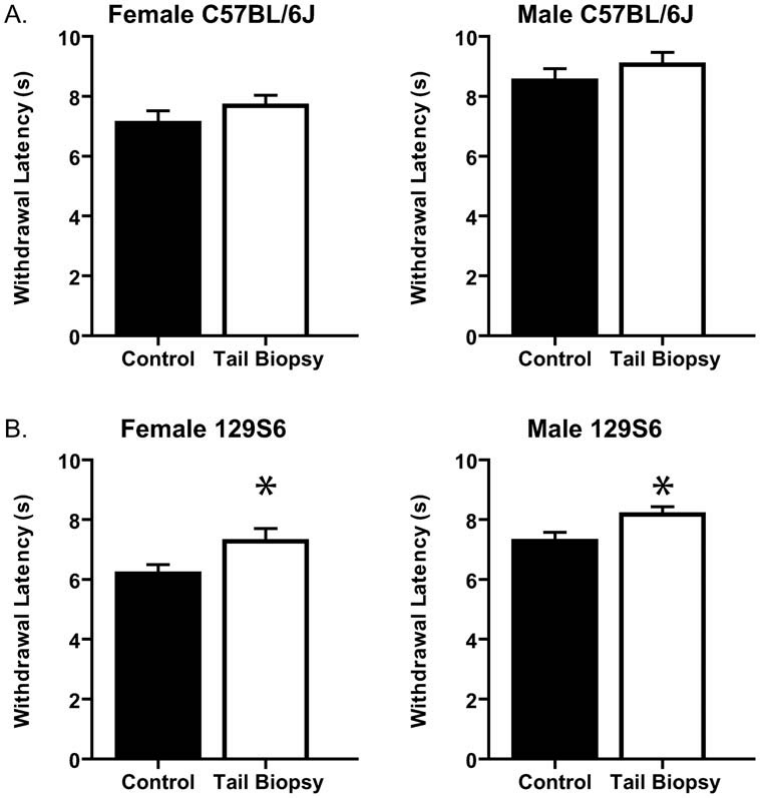
The effects of tail biopsy on thermal sensitivity. There are strain-specific differences in thermal sensitivity of tail-biopsied and control adult female and male mice. A. There are no significant differences in thermal sensitivity of female and male C57BL/6J mice as withdrawal latencies (s) of tail-biopsied and control female (left panel, n = 10 in both groups; age range: 6.14–6.86 w) and male mice (right panel, n = 11 tail biopsy and n = 12 control; age range: 6.14–6.86 w) do not differ (p = 0.3006 and 0.3515, respectively). B. There is a small but statistically significant difference in withdrawal latencies (s) of tail-biopsied and control female (left panel, n = 8 tail biopsy and n = 11 control; age range: 5.86–7.29 w) and male mice (right panel, n = 12 tail biopsy and n = 10 control; age range: 6.43–7.29 w) of the 129S6 strain (p = 0.048 and 0.0339, respectively).

As indicated by Zhuo (1998), thermal sensitivity of the hind paw can be altered by significant tail amputation as measured by the hot plate test. In order to determine the potential changes in thermal sensitivity due to tail biopsy representative of genotyping practices, we measured latency of withdrawal from radiant thermal heat applied to the plantar surface of the hind paw. In both female and male C57BL/6J mice, there were no significant differences in the withdrawal latencies of both hind paws to radiant heat stimulation (Fig. 2a). However, in 129S6 female and male mice, small but significant differences in paw withdrawal latency were observed between tail-biopsied and control mice (Fig. 2b).

### Mechanical sensitivity of the tail

**Figure 3:**
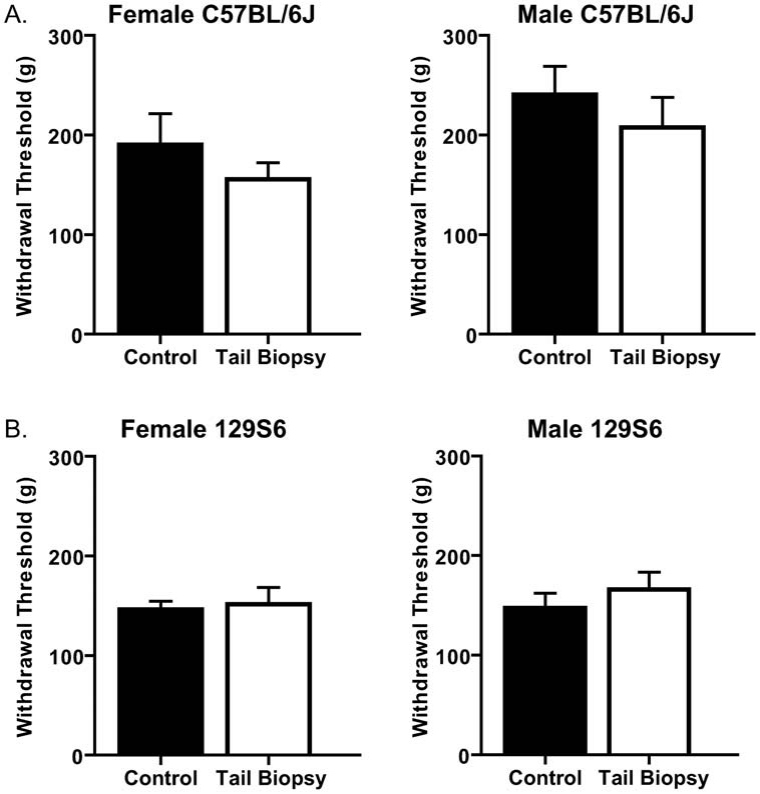
The effects of tail biopsy on tail mechanical sensitivity. There are no significant differences in tail mechanical sensitivity of tail-biopsied and control female and male mice of both C57BL/6J and 129S6 strains. A. Withdrawal thresholds (g) of tail-biopsied and control C57BL/6J female (left panel, n = 10 in both groups; age range: 6.14–8.86 w) and male mice (right panel, age range: 6.14–9.43 w) do not differ (p = 0.3354 and 0.4339, respectively). B. Withdrawal thresholds (g) of tail-biopsied and control 129S6 female (left panel, n = 8 tail biopsy and n = 11 control; age range: 5.86–7.29 w) and male mice (right panel, n = 12 tail biopsy and n = 10 control; age range: 6.43–7.29 w) also do not differ (p = 0.761and 0.443, respectively).

Since the primary site of injury in a tail biopsy is the not the hind paws, it was important to examine changes in behavioral responses near the injury location. A change in plasticity that results in alteration of behavioral responses might be more evident as one examines responses to stimuli closer to the site of injury. However, upon examination of tail withdrawal thresholds to mechanical pressure, no significant differences were observed in either the C57BL/6J or 129S6 female and male mice (Fig. 3a-b).

### Formalin test in C57BL/6J mice

**Figure 4:**
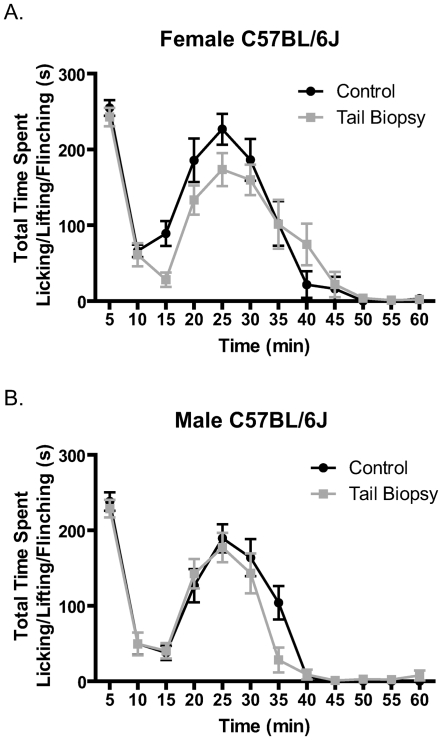
The effects of tail biopsy on response to formalin injection. There are no significant differences in spontaneous behavior elicited by injection of 2% formalin into the right hind paws of C57BL/6J female and male mice. Mice were placed in testing chambers for at least 2 h of acclimation prior to injection. Formalin (2%) was injected into the right hind paw and the time spent licking, lifting, and flinching was measured in 5 min blocks for 1 h after injection. Data were analyzed using a two-way repeated measures ANOVA. a) Time-course of spontaneous behavior following formalin injection indicates no difference in response between control and tail-biopsied female mice (n = 10 control and n = 12 tail biopsy; age range: 6.29–8.86 w). b) Time-course of spontaneous behavior following formalin injection indicates no difference in response between control and tail-biopsied male mice (n = 12 control and n = 11 tail biopsy; age range: 6.29–9.43 w).

Although mechanical and thermal baseline sensitivity are good measures of changes in nociceptive responses due to plasticity after tail biopsy, it is possible that alterations in behavioral responses could be more robust in the context of injury. Therefore, it was necessary to examine the differences in behavioral response to an injurious stimulus which results in inflammation. Because 129S6 mice lack a robust response in their formalin test [16], only C57BL/6J mice were tested. In C57BL/6J female and male mice, both tail-biopsied and control mice exhibited similar responses in the formalin test with no significant differences at any time point (Fig. 4a-b).

## Discussion

Tail biopsy for the purpose of genotyping is an integral tool in the use of genetically modified mice for studies of nociception. Although a previous report [8] indicated that tail amputation induced changes in behavioral responses to a number of tests of nociception, the methods used were not representative of standard genotyping protocols and were more characteristic of significant limb amputation. Here, we systematically examined the effects of tail biopsy, specifically for the purpose of genotyping, on nociception. Consistent with sex differences already described in the literature [21]–[24], we observed main effects of sex in both C57BL/6J and 129S6 strains in the Hargreaves test, in the 129S6 strain in the von Frey test of mechanical sensitivity, and in the C57BL/6J strain in the formalin test. In all tests where a main effect of sex was observed, female mice exhibited increased sensitivity as compared to male mice. In light of these differences and those already described in the literature, we separated data from male and female mice for analysis. We observed no significant differences between tail-biopsied and control female and male C57BL/6J mice in all tests performed (von Frey, Hargreaves, modified Randall-Selitto, and formalin). In addition, we observed no significant differences between tail-biopsied and control female and male 129S6 mice in mechanical sensitivity to non-noxious stimuli (von Frey test). Interestingly, both female and male 129S6 mice exhibited small and minimally significant (p = 0.048 and p = 0.0339 respectively) differences between tail-biopsied and control mice in their responses to a radiant heat stimulus (Hargreaves test). In both sexes, there was a slight increase in paw withdrawal latency with tail biopsy (0.88±0.39 s in males and 1.08±0.51 s in females). While this is statistically significant with a p-value<0.5, one might question the physiological relevance of a difference of this magnitude. Although a difference of ∼1 s in withdrawal latency to a thermal stimulus may be considered negligible, it is an important consideration when interpreting data from genetically modified mice. We do not know the mechanism underlying this change, but if one is studying a mutation that impacts pain-related behaviors and sensitization, it is possible that this small change in withdrawal latency could confound results and lead to invalid interpretations.

Several studies of early-life injury and subsequent pain-related behavior in adulthood have reported decreased thermal sensitivity in previously injured animals [4]–[7]. Interestingly, the presence of hypoalgesia appears to be dependent upon the intensity and duration of the injury. With a single injection of complete Freund's adjuvant (CFA), an inflammatory agent, at P0 resulting in inflammation lasting 24 h, animals exhibited thermal and mechanical *hypoalgesia* in adulthood [7], whereas animals receiving an injection of CFA at P0, which resulted in inflammation lasting up to 7 days, exhibited thermal *hyperalgesia* in adulthood [1]. In the present study, in mice receiving tail biopsies in early-life, we observed a small and minimally significant increase in withdrawal latency to a thermal stimulus when compared to responses of control animals. The injury induced by tail biopsy results in a short-term and spatially-restricted inflammation of the severed end of the tail. Thus, the difference in behavior seen here correlates with the intensity and duration of the injury in a manner consistent with previous reports.

Inconsistent with the previous data, however, is the timing of the injury with reference to development of the nervous system. In addition to the intensity and duration of the injury, the age of the animal at the time of injury is a critical factor in the development of alterations in pain-like behaviors. In rats, the sensitive period during which early-life injury can alter nociceptive thresholds in adulthood appears to end around P10 [1], [6], [7], [25]. These data suggest that in the present study where the injury was induced at P21 the sensitive period has likely ended.

It is important to note that the only difference observed in this study is in thermal sensitivity of the 129S6 strain. In C57BL/6J tail-biopsied and control mice of both sexes, no significant differences were observed in hind paw mechanical and thermal sensitivity, mechanical sensitivity of the tail, or formalin response. In 129S6 tail biopsied and control mice of both sexes, no significant differences were observed in hind paw and tail mechanical sensitivity. A small and minimally significant difference was observed in hind paw thermal sensitivity. This is consistent with previously described strain- and modality-specific differences in pain-related behaviors [26] and highlights the importance of the use of congenic strains and proper controls in analysis of these behaviors.

Taken together, the data presented here suggest that tail biopsy can be performed on mice with little or no impact on pain-related behaviors. Thus, for those who have avoided tail biopsy because of this potential confound, this information could significantly reduce the number of animals necessary for tests of nociception as investigators could genotype genetically modified mice prior to behavior testing and eliminate the animals whose genotype is not of interest.
